# Cross-Regional Comparative Study on Carbon Emission Efficiency of China’s Yangtze River Economic Belt Based on the Meta-Frontier

**DOI:** 10.3390/ijerph16040619

**Published:** 2019-02-20

**Authors:** Ze Tian, Fang-Rong Ren, Qin-Wen Xiao, Yung-Ho Chiu, Tai-Yu Lin

**Affiliations:** 1 School of Business Administration, Hohai University, Changzhou 213022, China; 20031655@hhu.edu.cn (Z.T.); xqw19951105@hhu.edu.cn (Q.-W.X.); 2 Business School, Hohai University, Nanjing 211100, China; 180213120008@hhu.edu.cn; 3 Department of Economics, Soochow University, Taipei 10048, Taiwan; eickyla@gmail.com

**Keywords:** EBM, meta-frontier, Yangtze River Economic Belt, YREB, CO_2_ performance, efficiency

## Abstract

The Yangtze River Economic Belt (YREB) is one of the most important areas for the economic growth of China, but rapid development has caused tremendous damage to the energy and ecological environments of the region. Very few studies have compared the carbon emissions of YREB with that of non-YREB and furthermore, have not considered regional differences and radial or non-radial characteristics in their analysis. This paper thus selects the energy consumption data of 19 provinces and cities in YREB and 19 provinces and cities in non-YREB from 2013 to 2016, constructs the modified meta-frontier Epsilou-based measure (EBM) data envelopment analysis (DEA) model and adds an undesirable factor, energy consumption, and CO_2_ emission efficiency of each province and city of the two regions. The results are as follows. (1) China’s provinces and cities have different energy efficiency scores in energy consumption, economic growth, and CO_2_ emissions. The regional ranks and technology gaps of five provinces and cities in non-YREB and of four provinces and cities in YREB exhibit a decline. Overall, the ranks and technology gaps of the provinces and cities in YREB are significantly lower than those in non-YREB, meaning that there is greater room for efficiency improvement in the latter region. (2) The gross domestic product (GDP) and CO_2_ efficiency values of non-YREB provinces present great differences, especially the CO_2_ efficiency value that ranges from 0.2 to 1, while their values in YREB are more balanced with little difference between provinces and cities. Thus, YREB is more coordinated in terms of energy savings and air pollutant reduction. (3) Some cities with good economic development such as Beijing, Shanghai, and Tianjin have regional and technology gap values of one, indicating that they not only target economic growth but also address energy savings and air pollutant reduction. The regional rank and technology gap values of some underdeveloped provinces such as Neimenggu, Ningxia, and Qinghai are also one. Finally, this research proposes countermeasures and recommendations to both areas.

## 1. Introduction

The Yangtze River Economic Belt (YREB) crosses east and west China and covers 11 provinces and municipalities in an area of about 2.05 million km^2^, which is 21% of the country’s land mass and 40% of its total population, making it one of the most comprehensively developed economic regions. According to People’s Daily of China, the annual cargo volume of the trunk line of the Yangtze River hit 2.69 billion tons in 2018, ranking first in the world in terms of inland rivers [1]. However, sustained and rapid economic growth has damaged the ecological environment of the region. Therefore, researching the relationships between energy use, industrial restructuring, and economic growth in YREB is significant for understanding China’s regional development. In fact, the China government’s main goal is “building a green energy industry belt along the Yangtze River” to make it “the pioneering demonstration belt for building an ecological civilization”.

There are four main directions in the field of energy consumption and low carbon economy research: (1) energy and CO_2_ emission reduction efficiency [2,3,4,5,6,7]; (2) energy and environmental efficiency assessments for different countries and industries [8,9,10,11,12,13,14,15]; (3) GDP growth and energy analysis [16,17,18]; (4) efficiency analysis of energy, CO_2_, and SO_2_ (or NO_2_) [19,20,21,22,23,24,25]. However, these studies in the literature lack analysis of energy consumption and low carbon in YREB.

This present paper, therefore, has the following three major innovations and contributions. First, China’s energy and environment research at the regional level mainly focuses on the division of provinces and cities, as well as the traditional three regions (eastern, central, and western). In September 2014, the State Council of the People’s Republic of China issued guiding opinions on promoting the development of YREB by relying on the “golden waterway” and proposed the division of YREB for the first time. At present, no scholars in China have ever evaluated the energy and environmental efficiencies of YREB and non-YREB. As the middle and lower reaches of the Yangtze River are the most developed areas in China, comparing the energy efficiency of YREB with that of other regions can provide a new division basis and evaluation results for the country’s regional economic development and help provinces to adjust or optimize their industrial structure.

Second, YREB straddles the two major regions of east and west China, and its population density, economic density, and per capita GDP are respectively 4.5, 6.2, and 1.4 times the national average. Having the largest economic density in China, its strategic significance for China’s economic development is incomparable with that of other economic belts in the world. In fact, it is the region with the largest economic growth potential in the next 15–20 years and should become an inland river economic belt with the largest development scale and widest influence scope globally. Therefore, the findings herein benefit not just China, but also other important river basins’ economic belt research.

Third, the research findings of this paper can be used to further verify whether energy conservation and emission reduction are ignored or paid greater attention to during the process of strong economic growth. Moreover, we shall see where the carbon emission efficiency of the more developed YREB is versus the less developed non-YREB.

Fourth, as current research generally uses DEA, the main methods employed are radial analysis, such as CCR (Charnes, Cooper, Rhodes) and BCC (Banker, Charnes, Cooper), or non-radial analysis, such as SBM (slacks-based model) or DDFM (directional distance function model). However, the radial DEA model ignores non-radial slacks, while the non-radial DEA model ignores the characteristics of the same proportion of radial DEA model. The EBM model is a combination of radial and non-radial DEA model. Thus, we select the EBM DEA model to avoid an underestimation or overestimation of efficiency values and improvement space.

Our research modifies the EBM model of Tone and Tsutsui [26], adds undesirable variable factors and common boundaries and proposes a modified meta-frontier Undesirable EBM DEA model to evaluate the energy and pollution of 30 provinces and cities in YREB and non-YREB. China is the world’s largest energy consumer and carbon emissions country, and the YREB and non-YREB regions have major regional disparities. In this context, if the traditional ray or non-radiation DEA method is used, then the energy and carbon emission efficiency index calculation results will be biased. We hence divide the sample data into YREB and non-YREB to eliminate regional differences and production estimation errors. Our main contribution is using the undesirable EBM DEA method to avoid any underestimation or overestimation of the efficiency caused by radial and non-radial DEA.

The organization of this paper runs as follows. Section 2 is a literature review. Section 3 covers the research method. Section 4 presents the empirical results and discussions. Section 5 is the conclusions.

## 2. Literature Review

Research on energy, environment, and low-carbon economy has typically focused on CO_2_ emission reduction efficiency in recent years. Pao et al. [2] have examined the dynamic causal relationships among pollutant emissions. Yao et al. [3] have looked at CO_2_ emissions, analyzing countryside energy consumption in China during 2001–2008 and the effects on climate change. Yao et al. [4] considered heterogeneity, divides carbon emissions into two parts—fossil fuel carbon emissions and non-fossil fuel carbon emissions—and analyzed the impact of technology gaps on regional energy efficiency and carbon emission performance in China. Yao et al. [5] used the meta-frontier non-radial Malmquist CO_2_ emission performance index (MNMCPI) to estimate carbon dioxide emission efficiency in China. The results show from 1998 to 2011 that its provincial industrial sector carbon dioxide emissions grew at an average annual rate of 5.53%, while the average carbon dioxide emissions of the industrial sectors in the eastern, central, and western regions fell. Du et al. [7] estimated potential energy intensity (PEI) changes, energy structure changes (ESC) and energy efficiency changes (EEC) from 2006 to 2012 in order to reduce CO_2_ emissions in most provinces/cities in China. Decreasing inter-regional technology differences can effectively cut regional carbon dioxide emissions.

Some scholars have employed the DEA method to evaluate energy and environmental efficiencies in different countries and industries. Blomberg et al. [8] evaluated the pulp and paper industry’s electricity efficiency improvement in Sweden by DEA and mill-specific input and output in 1995, 2000, and 2005. Bian et al. [9] assessed energy efficiencies in different regions of China with a non-radial DEA model. Zhang et al. [10] researched electricity generation energy efficiency and CO_2_ emissions by a non-radial directional distance function meta-frontier. Pang et al. [11] used SBM to evaluate the efficiency of 87 countries and find that European countries have more efficiency in reducing emissions and saving energy. Li and Lin [12] utilized a meta-frontier framework to reflect the heterogeneous technology of eastern, central, and western China. Suzuki et al. [13] developed a target-oriented distance function model (TODFM) and super-efficiency DEA, exploring that the efficiency performances in EU countries are better than those in APEC and ASEAN countries. Guo et al. [14] employ SBM Dynamic DEA to analyze energy efficiency and find that 27 countries’ energy efficiency is improving. Wang et al. [15] took the meta-frontier function and the non-radial DEA method to analyze the carbon emissions of 58 countries from 2001 to 2007 and show that Asia’s overall carbon emissions are lower than those in Europe and America. Low management efficiency and production technology gaps are the two main reasons.

Some scholars have researched the relationship between GDP growth and energy consumption. Niu et al. [16] evaluated the energy consumption, GDP growth, and carbon emissions causality of eight Pacific countries in Asia during 1971–2005. Zhang et al. [17] considered that greater GDP causes the largest increase in carbon emissions, whereas falling energy intensity significantly decreases emissions. Atems and Hotaling [18] used the system generalized method of moments (GMM) of 174 countries from 1980 to 2012 to estimate the economic impact of power generation, presenting a significant relationship between renewable energy and non-renewable energy generation and economic growth.

Some studies have considered CO_2_ and SO_2_ (or NO_2_) together and regard them as undesirable outputs for energy environment efficiency assessment. Tsolas [19] uses DEA to explore the capacity efficiency of fossil fuel power plants in Greece. Sampling a 50-megawatt (MW) fossil fuel power plant there in 2004, the input variables are the operation time of the power station and the number of employees, and the variable net power is the intended output; SO_2_, NO_X_, and CO_2_ are unintended outputs. Inefficient power plants are less efficient than the initial estimation, while non-lignite plants are on average more efficient than power plants. Sueyoshi and Mika [20] analyzed the effects of the U.S. Clean Air Act (CAA) on acid-induced gas (NO_X_) by a non-radial DEA model and measure the environmental performance of plants under two alternatives (with or without CO_2_ emission control). Under the CAA specification, coal-fired power plants in the U.S. are efficient at controlling SO_2_ and NO_X_ emissions.

Li and Hu [21] considered CO_2_ and SO_2_ emissions and calculate the ecological total-factor energy efficiency (ETFEE) of 30 regions in China from 2005 to 2009 by the SBM model. China’s regional ETFEE still maintains roughly 0.6, while regional energy efficiency is estimated to exceed 0.100 regardless of the environmental impact. The regional energy efficiency in China is extremely uneven: the eastern region ranks first with ETFEE higher than 0.700, followed by the northeast and central regions, while the western region has the lowest ETFEE at below 0.500. Wang and Wei [22] used DDFM to analyze the energy efficiency of China and explore that energy efficiency has significant growth in carbon dioxide emissions. Wang and Feng [23] took the SBM model and focus on China’s energy, environment, and economy for efficiency and productivity analyses. The results show that China’s economy has performed well, while energy and the environment have performed poorly, but in recent years, energy and environmental efficiencies have gradually increased.

Qin et al. [24] used the Malmquist-Luenberger Productivity Index to assess the coastal areas’ energy efficiency in China during 2000–2012, in which CO_2_, SO_2_, and NO_X_ are considered as undesirable outputs. Except for Beijing and Hainan, the energy efficiencies have declined. Improving energy efficiency depends primarily on technological improvements, economies of scale, and management. Guo et al. [25] utilized total factor energy efficiency to assess coal consumption efficiency in six energy-intensive industries of China in 2015, where sulfur dioxide, nitrogen oxides, and industrial smog, dust, and soot emissions are considered as undesirable outputs. The results show that only two of the six energy-intensive industries are efficient, and so China should pay more attention to cutting down on the use of coal.

According to the above literature, there is currently no specific energy and CO_2_ efficiency assessment of the YREB and non-YREB regions in China. While the main methods in the literature are radial or non-radial models, they may underestimate or overestimate production efficiency values. Therefore, this paper adopts the modified meta-frontier Undesirable EBM DEA to assess energy efficiency and CO_2_ efficiency in 30 provinces and cities of China, in order to reflect carbon emission efficiency more objectively and accurately.

## 3. Materials and Methods 

Charnes et al. [27] set up the CCR DEA model based on Farrell’s [28] concept of a “frontier”. Banker et al. [29] extended its assumptions on scale returns and propose the BCC model. As CCR and BCC are radial DEA models, they ignored non-radial slacks when evaluating efficiency value. Thus, Tone [30] proposed SBM and presents a non-radial estimation with a single scalar. SBM is a non-radial DEA model and did not consider the characteristics of the radial model. In order to solve the shortcomings of the radial and non-radial models, Tone and Tsutsui [26] set up the EBM DEA model because the three methods of input orientation, output orientation, and non-orientation can solve the shortcomings of the radial DEA and non-radial DEA models. 

We present now the Tone and Tsutsui [26] EBM DEA basic model and solution.

The total number of DMUs is *n*, where $DMU_{j}=\left(DMU_{1},DMU_{2},\ldots \ldots ,DMU_{k},\ldots \ldots DMU_{n}\right)$, there are *m* types of input $X_{j}=\left(X_{1j},X_{2j},\ldots \ldots ,X_{mj}\right)$, and the number of outputs is *s*, where $Y_{j}=\left(Y_{1j},Y_{2j},\ldots \ldots ,Y_{sj}\right)$.

The efficiency of the DMU unit is:

$$K^{*}=\underset{0,\eta ,\lambda ,s^{-},s^{+}}{\min}\frac{\theta -\varepsilon_{x}\sum_{i=1}^{m}\frac{w_{i}^{-}s_{i}^{-}}{x_{i0}}}{\eta +\varepsilon_{y}\sum_{i=1}^{s}\frac{w_{i}^{+}s_{i}^{+}}{y_{i0}}}$$

(1) $$\mathrm{Subject}\;\mathrm{to}\;\theta X_{0}-X_{\lambda }-S^{-}=0,$$

$\eta Y_{0}-Y_{\lambda }+S^{+}=0$,

$\lambda_{1}+\lambda_{2}+\ldots +\lambda_{n}=1$

$\lambda \geq 0,\;S^{-}\geq 0,\;S^{+}\geq 0$.

Y: DMU output,
X: DMU input,
$S^{-}$: slack variable,
$S^{+}$: surplus variable,
$W^{-}$: weight of input i, $\sum W_{i}^{-}=1\;\left(\forall_{i}W_{i}^{-}\geq 0\right)$,
$W^{+}$: weight of output s, $\sum W_{i}^{+}=1\;\left(\forall_{i}W_{i}^{+}\geq 0\right)$,
$\varepsilon_{x}$: a set of radial θ and non-radial slack,
$\varepsilon_{y}$: a set of radial η and non-radial slack.

For DMU0, when $K^{*}=1$, EBM is the most non-oriented efficient. If the DMU is inefficient, then the following adjustments are needed:

$$X_{0}^{*}=X\lambda^{*}=\theta^{*}X_{0}-S^{-*}$$

$$Y_{0}^{*}=Y\lambda^{*}=\eta^{*}y_{0}+S^{+}$$

### 3.1. The Research Model: Modified Meta-Frontier EBM DEA Model

Tone and Tsutsui [26] proposed the EBM (Epsilou-Based Measure) DEA model, including input-oriented, output-oriented, and non-oriented, which can solve the disadvantages of the radial DEA model and the non-radial DEA model. DEA generally assumes all producers have homogeneous technology, however, DMUs, in reality, have technology differences due to geographical locations, government policies, economy, etc. Battese and Rao [31] and Battese et al. [32] proved that the meta-frontier method can help compare the technical efficiency (TE) of each group. O’Donnell et al. [33] explored that the meta-frontier method can evaluate the technology efficiency of each group and meta-frontier.

Tone and Tsutsui’s [26] EBM does not limit the range of variables θ and η and does not consider any undesirable factor. This paper considers different types of management, resources, regulations, and environments and presents the modified meta-frontier EBM DEA Model to assess the energy efficiency of 30 provinces and cities in China. Our target is to avoid efficiency values being underestimated or overestimated as caused by regional differences. The model runs as follows. 

Due to differences in management, resources, regulations, and environments, we compose all firms (*N*) by groups of DMUs (*N* = *N*_1_ + *N*_2_ +…+ *N*_G_), and $x_{ij}$ and $y_{rj}$ respectively denote input *i* (*i* = 1, …, *m*) and final output *r* (*r* = 1, …, *s*) of the DMU unit *j* (*j* = 1, …, *N*). Under the meta-frontier, DMU *k* can choose the weight of the final output $u_{r}^{g}\left(r=1,\ldots ,s\right)$ to achieve the biggest efficiency value. Therefore, under non-oriented EBM, we can solve the efficiency of DMU k under the meta-frontier by the following linear programming procedure.

The number of DMUs is *n*, where $DMU_{j}=\left(DMU_{1},DMU_{2},\ldots \ldots ,DMU_{k},\ldots \ldots DMU_{n}\right)$. There are *m* types of input $X_{j}=\left(X_{1j},X_{2j},\ldots \ldots ,X_{mj}\right)$, and the number of outputs is *s*, where $Y_{j}=\left(Y_{1j},Y_{2j},\ldots \ldots ,Y_{sj}\right)$. The efficiency of the DMU unit is:

$$K^{*}=\underset{0\eta ,\lambda ,s^{-},s^{+g},s^{-b}}{\min}\frac{\theta -\varepsilon_{x}\sum_{g=1}^{G}\sum_{i=1}^{m}\frac{W_{i}^{-}S_{i}^{-}}{X_{i0}}}{\eta +\varepsilon_{y}[\sum_{g=1}^{G}\sum_{i=1}^{S1}\frac{w_{i}^{+S1}s_{i}^{+good}}{y_{i0}}+\sum_{i=1}^{S2}\frac{w_{i}^{-S2}s_{i}^{-bad}}{y_{i0}}]}$$

(2) $$\begin{array}{c} \mathrm{Subject}\;\mathrm{to}\;X_{i0}=\sum_{g=1}^{G}\sum_{j=1}^{n}X_{ijg}\theta_{jg}-S_{i}^{-}\;\left(i=1\ldots m\right)\\ Y_{i0}=\overset{G}{\underset{g=1}{\sum }}\overset{n}{\underset{j=1}{\sum }}Y_{ijg}^{+good}\eta_{jg}+S_{i}^{+good}\;\left(i=1\ldots s\right)\\ Y_{i0}=\overset{G}{\underset{g=1}{\sum }}\overset{n}{\underset{j=1}{\sum }}Y_{ijg}^{-bad}\eta_{jg}-S_{i}^{-bad}\;\left(i=1\ldots s\right)\\ \overset{G}{\underset{g=1}{\sum }}\overset{n}{\underset{j=1}{\sum }}\lambda_{jg}=1 \end{array}$$

$\lambda \geq 0,S^{-}\geq 0,S^{+g}\geq 0,S^{-b}\geq 0$, θ≤1, η≥1

Y: DMU output,
X: DMU input,
$S^{-}$: slack variable,
$S^{+g}$: surplus variable,
$S^{-b}$: surplus variable,
$W^{-}$: weight of input i, $\sum W_{i}^{-}=1\;\left(\forall_{i}\;W_{i}^{-}\geq 0\right)$,
$W^{+}$: weight of output S, $\sum W_{i}^{+S1}+\sum W_{i}^{-S2}=1\;\left(\forall_{i}\;W_{i}^{+}\geq 0\right)$
$\varepsilon_{x}$: a set of radial θ and non-radial slack,
$\varepsilon_{y}$: a set of radial η and non-radial slack.

From the above equations, we find the overall technical efficiency values of manufacturers under the meta-frontier.

#### 3.1.1. Weighted SBM Dynamic Group Frontier Model

We took the 30 provinces in China and divide them into g groups of DMUs, where the DMU under each group frontier chooses the optimal final output weight. Therefore, we solved the DMU efficiency under the group frontier by:

$$K^{*}=\underset{0\eta ,\lambda ,s^{-},s^{+g},s^{-b}}{\min}\frac{\theta -\varepsilon_{x}\sum_{i=1}^{m}\frac{w_{i}^{-}s_{i}^{-}}{x_{i0}}}{\eta +\varepsilon_{y}[\sum_{i=1}^{S1}\frac{w_{i}^{+S1}s_{i}^{+g}}{y_{i0}}+\sum_{i=1}^{S2}\frac{w_{i}^{-S2}s_{i}^{-b}}{y_{i0}}]}$$

(3) $$\begin{array}{c} \mathrm{Subject}\;\mathrm{to}\;\theta X_{0}-X_{\lambda }-S^{-}=0,\\ \eta Y_{0}-Y_{\lambda }^{+g}+S^{+g}=0\\ \eta Y_{0}-Y_{\lambda }^{-b}-S^{-b}=0\\ \lambda_{1}+\lambda_{2}+\ldots +\lambda_{n}=1 \end{array}$$

$\lambda \geq 0,S^{-}\geq 0,S^{+g}\geq 0,S^{-b}\geq 0$, θ≤1, η≥1

Y: DMU output,
X: DMU input,
$S^{-}$: slack variable,
$S^{+g}$: surplus variable,
$S^{-b}$: surplus variable,
$W^{-}$: weight of input i, $\sum W_{i}^{-}=1\;\left(\forall_{i}\;W_{i}^{-}\geq 0\right)$
$W^{+}$: weight of output S,$\;\sum W_{i}^{+S1}+\sum W_{i}^{-S2}=1\;\left(\forall_{i}\;W_{i}^{+}\geq 0\right)$
$\varepsilon_{x}$: a set of radial θ and non-radial slack,
$\varepsilon_{y}$: a set of radial η and non-radial slack.

#### 3.1.2. Technology Gap Ratio (TGR)

The production frontier of the g groups was included in the meta-frontier. Here, the technical efficiency under the group frontier must be bigger than under the meta-frontier. The ratio of the two frontiers is called (the technology gap ratio (TGR):

(4) $$\mathrm{TGR}=\frac{\rho^{*}}{\rho_{0}^{*g}}$$

### 3.2. Energy, GDP, and CO_2_ Efficiency Indices

Hu and Wang’s [34] total-factor energy efficiency index helps overcome any possible bias in any traditional energy efficiency indicator. For each specific evaluated country, we calculate its GDP, energy consumption, and CO_2_ efficiencies using Equations (5)–(7).

(5) $$\mathrm{GDP}=\frac{\mathrm{Actual}\;\mathrm{GDP}\;\mathrm{desirable}\;\mathrm{output}\;\left(i,\;t\right)}{\mathrm{Target}\;\mathrm{GDP}\;\mathrm{desirable}\;\mathrm{output}\;\left(i,\;t\right)}$$

(6) $$E\;=\;\frac{\mathrm{Target}\;\mathrm{energy}\;\mathrm{input}\;\left(i,\;t\right)}{\mathrm{Actual}\;\mathrm{energy}\;\mathrm{input}\;\left(i,\;t\right)}$$

(7) $${\mathrm{CO}}_{2}=\frac{\mathrm{Target}\;CO_{2}\;\mathrm{Undesirable}\;\mathrm{output}\;\left(i,\;t\right)}{\mathrm{Actual}\;CO_{2}\;\mathrm{Undesirable}\;\mathrm{output}\;\left(i,\;t\right)}$$

The GDP index is the most efficient when Equation (5) equals 1. On the contrary, the GDP index is inefficient when Equation (5) is less than 1. If Equations (6) and (7) equal 1, then the energy and CO_2_ indices are efficient. If Equations (6) and (7) are less than 1, then the energy and CO_2_ indices are inefficient. 

### 3.3. Data Sources and Description

From past research on energy and environment the inputs generally are labor, fixed assets, and energy consumption, such as in Hu and Wang [34], Li et al. [21], Wang and Wei [22], Du et al. [7], and Guo et al. [14], while outputs are mainly GDP and CO_2_, such as in Li et al. [21], Wang and Wei [22], Du et al. [7], and Guo et al. [14]. We employed data from 30 provinces and cities in China spanning 2013 to 2016. Data such as capital stock, labor force, and GDP were derived from the China Statistical Yearbook [35], and energy consumption and CO_2_ emissions are derived from the China Energy Statistics Yearbook [36]. The regional division of YREB and non-YREB comes from the “Guiding Opinions on Promoting the Development of the Yangtze River Economic Belt on the Golden Waterway” issued by the State Council of the People’s Republic of China in September 2014 [37]. The variables used herein are labor, fixed assets, new energy consumption, and energy consumption as input, GDP as output, and CO_2_ as undesirable output (see Table 1).

Input variables:

Labor input (lab): This study uses the number of employees in each region at the end of each year; unit is a person. 

Capital input (assets): We take capital stock according to the fixed asset investments in each city; the unit is 100 million RMB. Fixed asset investments in China Statistical Yearbook mainly refer to the total amount of construction projects, the purchase of fixed assets, and related expenses in the form of currency in each province within a certain period. Therefore, we selected this amount as an input index to reflect the cost of equipment and tools, construction and installation engineering, engineering construction and other costs invested in economic and social development.

Energy consumption (com): This covers each city’s total energy consumption; the unit is 100 million tons. New energy (com) includes solar energy, nuclear energy, and wind power. 

Consumption (com): We calculate this from each city’s total energy consumption; the unit is 100 million tons.

Output variable:

GDP: It commonly refers to the total value of all final products and services produced within a certain period of time (a quarter or a year) in the economy of a country or region. It can reflect both a country’s not only the economic performance and it is of a country but also the national strength and wealth of a country. At present, China calculates quarterly GDP data on a quarterly basis and publishes the final verified figures in the China Statistical Yearbook every September. The GDP from each province (city) is applied as the city’s output; unit is 100 million RMB. 

Undesirable output variable:

CO_2_ (carbon dioxide): It encompasses air emissions due to energy consumption.

### 3.4. Research Hypotheses 

Based on the previous research results and reasonable research assumptions, we formulated four research hypotheses that must be verified. First, we shall evaluate the energy and environmental efficiencies of YREB and non-YREB. Second, provinces (cities) have clearly not paid enough attention to energy conservation and emission reduction during economic growth, and their carbon emission efficiencies are not ideal [14]. Third, under GDP growth and increased investment in fixed assets and employment, provinces (cities) must focus on energy conservation and emission reduction, which will help carbon emission efficiency to rise [21]. Fourth, there is no obvious correlation between economic growth and carbon emission efficiency, and this study cannot explain the correlation between the two [4].

## 4. Results and Discussion

### 4.1. Statistical Analysis

**Figure 1:**
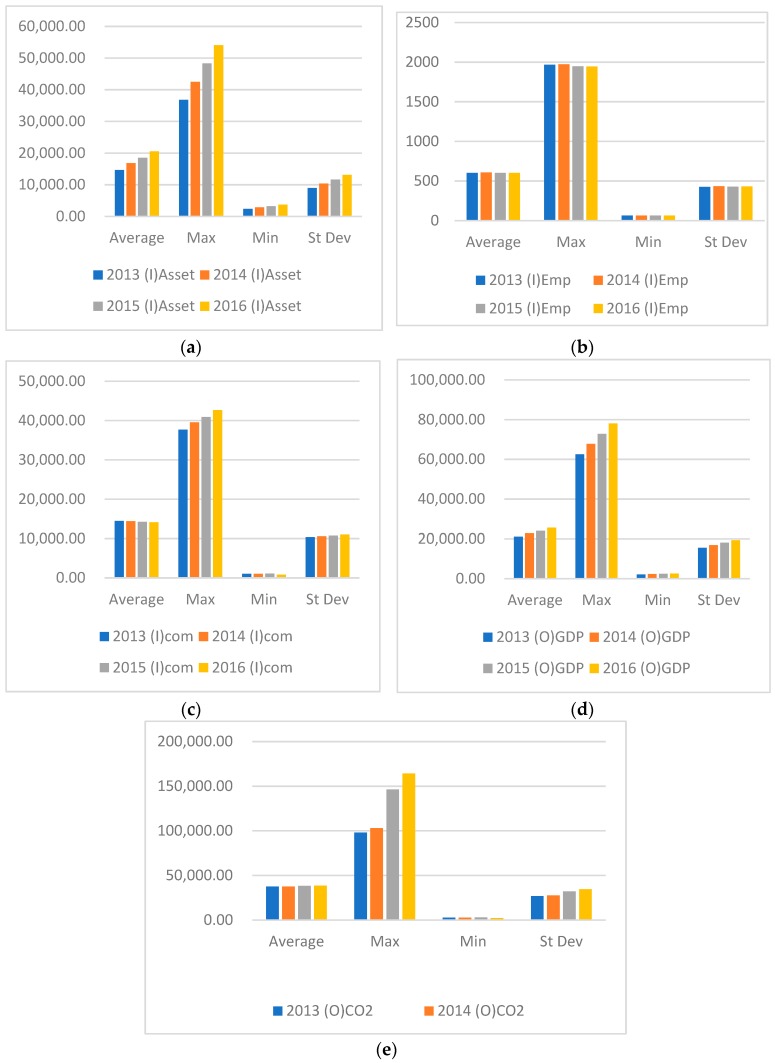
Statistical description of input and output variables by year. (Notes: The data source is References [35,36]). (**a**) Statistical description of Asset (input); (**b**) Statistical description of employees (input); (**c**) Statistical description of Energy consumption (input); (**d**) Statistical description of GDP (output); (**e**) Statistical description of CO2 (output).

Figure 1 shows the statistical data of fixed assets, employed population, traditional energy consumption, gross domestic product, and CO_2_ emissions. Among the three input factors, fixed assets continue to rise each year, and the maximum and the average number of employed people slightly decline since 2014. The average value of traditional energy consumption has dropped slightly, but the maximum value continues to rise.

Among outputs the average value of GDP has maintained a steady upward trend, the maximum value of GDP has increased significantly, and the minimum value has risen slowly. The increase in the average value of CO_2_ is not significant. It rises sharply in 2015, but declines in 2016. Most provincial municipalities and autonomous regions have begun to address CO_2_ emission reduction in their pursuit of GDP growth.

### 4.2. Overall Efficiency Score Ranking from 2013–2016

Table 2 shows the efficiency score and rank of each province from 2013 to 2016 (blue marks YREB provinces; the others are non-YREB provinces). The overall efficiency score of Shanghai in YREB and those of Beijing, Inner Mongolia, and Tianjin in non-YREB are 1 for four consecutive years. The analysis runs as follows.

From the provinces in YREB, Jiangsu, Hunan, Chongqing, Anhui, and Guizhou have scores and ranks that are stable. Sichuan and Yunnan show an obvious upward trend. Sichuan has increased by 7 in ranking in 2016 compared to 2013, and its overall efficiency value has gone from 0.7285 to 0.7879. Hubei and Jiangxi exhibit a significant decline.

From the provinces in non-YREB, their overall efficiencies are mostly all stable. Liaoning has the largest increase, with an efficiency value of 1 in 2016, which rises 10 in ranking versus 2013. Hainan shows a significant decline, from 0.8223 in 2013 to 0.7384 in 2016, or from 9th to 14th. There are also three provinces that have remained unchanged at the end of the ranking: Xinjiang, Shanxi, and Gansu.

Table 3 shows the energy consumption, GDP, and CO_2_ efficiency scores of different provinces during 2013–2016. Shanghai, Sichuan, and Zhejiang in YREB and Beijing, Tianjin, Fujian, Guangxi, and Hainan in non-YREB have higher efficiency scores. In particular, Shanghai, Beijing, and Tianjin have efficiency values of 1 in each year. There are some provinces and cities with large differences in GDP, energy consumption, and CO_2_ efficiency scores, such as Anhui, Guizhou, and Yunnan in YREB and Ningxia, Shanxi, and Xinjiang in non-YREB. These provinces and cities have higher GDP scores, all above 0.8, but their energy consumption and CO_2_ efficiency scores are all very low at under 0.27. For Shanxi, the difference between GDP and CO_2_ efficiency scores is above 0.8, showing a huge imbalance. All these provinces are targeting GDP growth, but there is still room for improvement in energy savings and pollution reduction.

**Figure 2:**
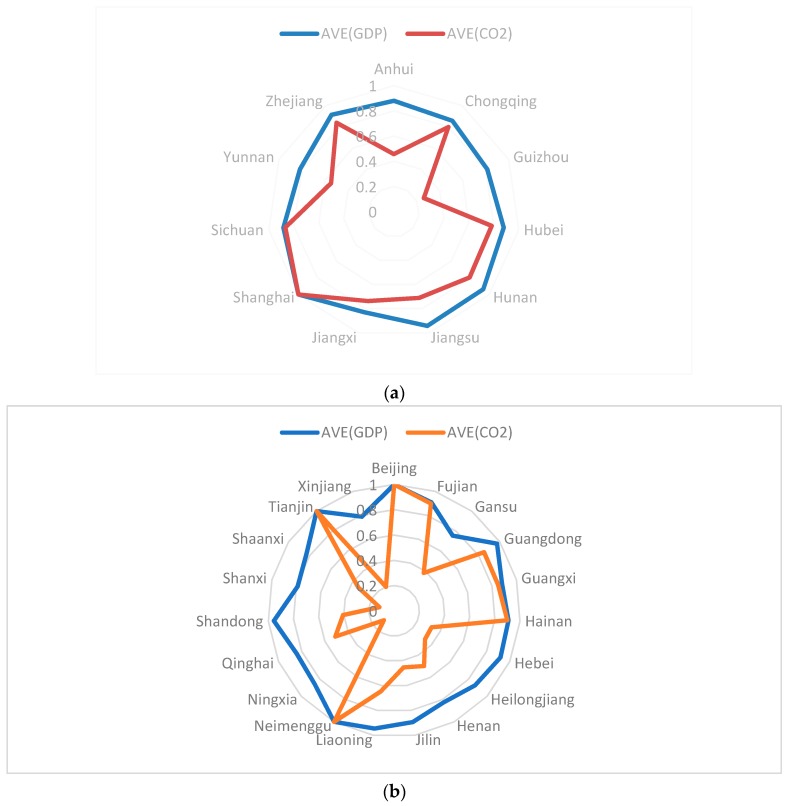
(**a**) GDP and CO_2_ efficiency scores of the Yangtze River Economic Belt. (**b**) GDP and CO_2_ efficiency scores of the non-Yangtze River Economic Belt. Notes: The data come from the authors’ collection.

From Figure 2, the GDP and CO_2_ efficiency values of YREB are more balanced, and the differences among provinces are small. However, there is a significant difference in the GDP and CO_2_ efficiency values of non-YREB provinces, especially as the CO_2_ efficiency value has a wide range from 0.2 to 1. Any improvement in the environment and efficiency of YREB will help China maintain its path of ecological priority and green development and offer a solution for environmental governance and sustainable development of the world’s large river basins.

**Figure 3:**
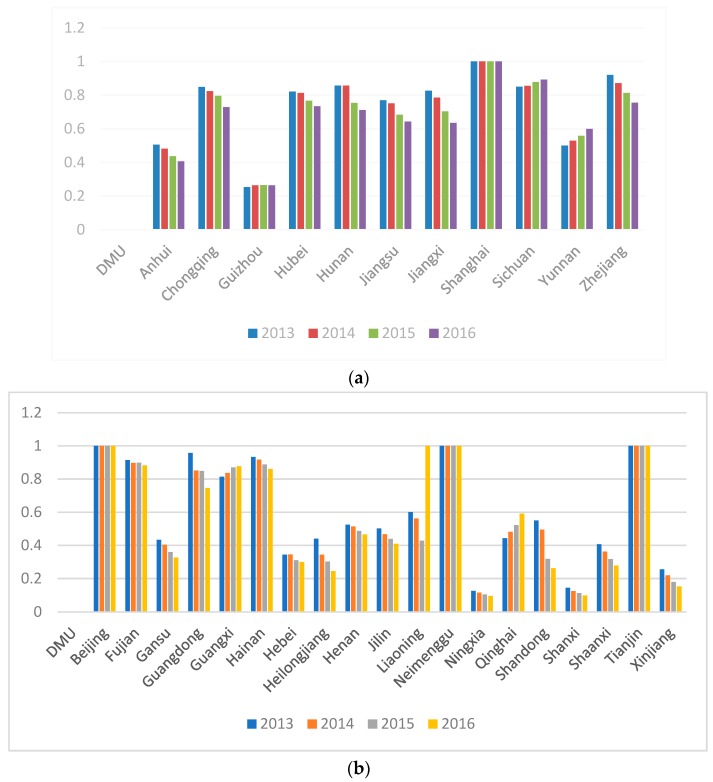
(**a**) CO_2_ efficiency scores of the Yangtze River Economic Belt. (**b**) CO_2_ efficiency scores of the non-Yangtze River Economic Belt. Notes: The data are from the authors’ collection.

From Figure 3, the CO_2_ emission efficiency values of YREB are relatively balanced between 0.4 and 0.8. However, the CO_2_ emission efficiency values of non-YREB show a large difference of between 0.1 and 1.

Table 4 lists the regional rank and technology gap values of each province during 2013–2016. The rank and technology gaps of non-YREB are significantly better than those of YREB. Moreover, 8 provinces and cities in YREB rank in the bottom ten in 2016. The technology gaps of YREB provinces are lower than those of non-YREB provinces from 2013 to 2016. In particular, Jiangsu ranks 29th in 2013 and rises to 24th in 2016. However, it is still a province with a low technology gap, which is related to its concentrated manufacturing industry with high energy consumption and low emission reduction efficiency.

The regional ranks of most provinces in YREB have not changed much. Shanghai has a technology gap of 1 and ranks 1st. Jiangxi and Hubei show a slight decline. Zhejiang experienced a very significant decline from 13th in 2013 to 19th in 2016 and thus needs to strengthen carbon dioxide emission treatment.

In the non-YREB regions, two provinces stand out. First, Liaoning’s technology gap values and ranks for 2013, 2014, and 2016 are all 1, but there is a fluctuation in 2015 when its technology gap value drops to 0.9594, and its rank declines to 20th. The other province is Guangxi, which jumps from 9th in 2013 to 1st in 2016. Its technology gap increases from 0.9973 to 1, indicating that its CO_2_ emission control has improved.

**Figure 4:**
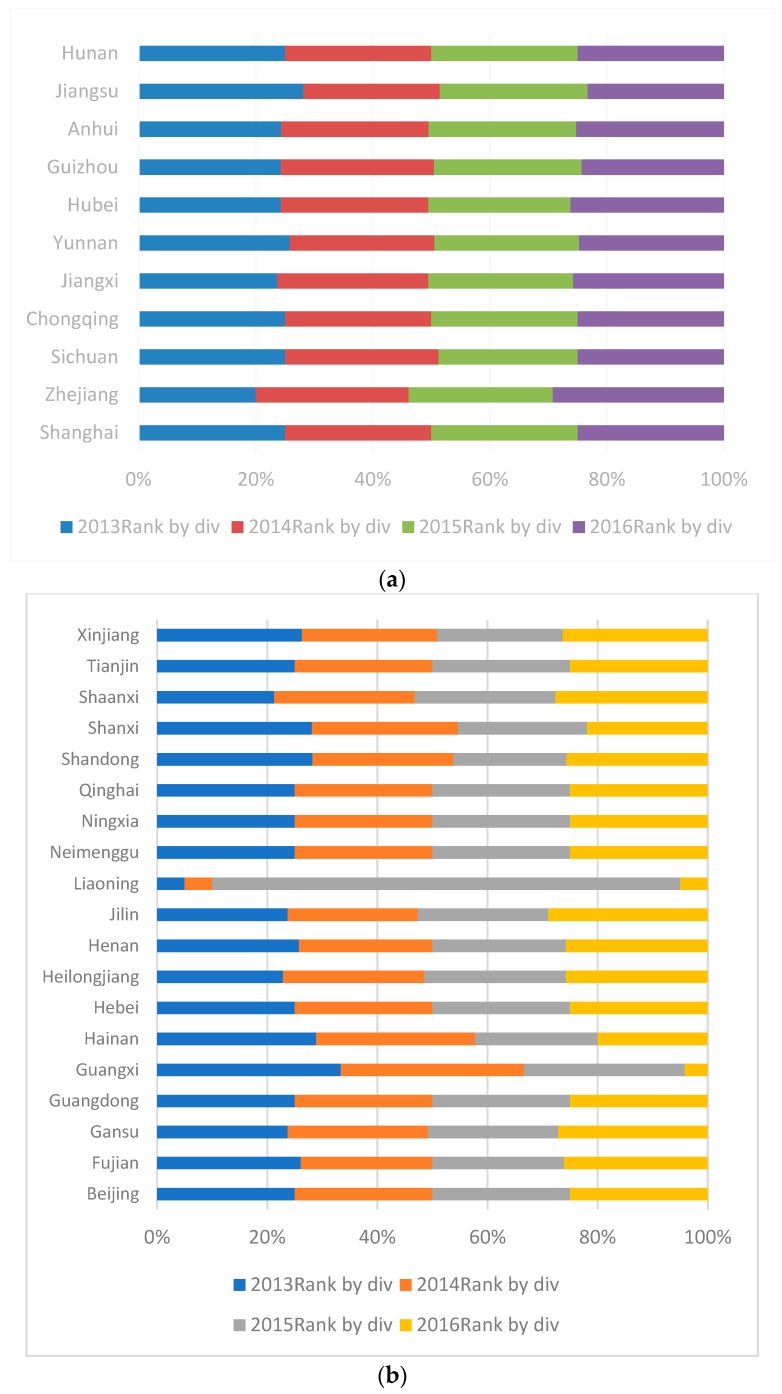
(**a**) 2013–2016 Yangtze River Economic Belt efficiency ranking. (**b**) Non-Yangtze River Economic Belt efficiency ranking. Notes: The data are from the authors’ collection.

Figure 4 shows the regional ranks during 2013–2016. The ranks of provinces in YREB are stable, indicating that there is no significant adjustment in CO_2_ emissions and treatment. The ranks of provinces in non-YREB are highly volatile, indicating that their policies are not stable enough in terms of CO_2_ emissions and treatment.

Table 5 shows the Wilcoxon test score for the average technology gap. In 2013–2016, the average gap of YREB and the average gap of non-YREB both pass the significance test.

### 4.3. Discussion of the Results

According to the above analysis, the CO_2_ efficiency values of provinces outside YREB exhibit large differences, and the prominent polarization is basically consistent with the research results of other scholars [6,7]. However, the ranking and technology gap values of provinces and cities in YREB are significantly behind those in non-YREB, which is slightly different from the current situation of some provinces that are considered low-carbon pilots [38]. It shows that while the YREB region focuses on rapid economic growth, more efforts and improvements are needed to provide non-YREB with experience that can be used for reference, copied, and popularized.

## 5. Conclusions

This research investigates 30 provinces and cities in China from the YREB and non-YREB regions to explore the correlations among energy consumption, CO_2_ emission efficiency, and economic performance in 2013–2016. The results are as follows. 

1. According to the meta-frontier, five provinces and cities in non-YREB (Jilin, Gansu, Shanxi, Heilongjiang, and Guangdong) and four provinces and cities in YREB (Zhejiang, Jiangxi, Hubei, and Anhui) decline in rank and technical gap. The proportion of provinces and cities with both a declining rank and technology gap in YREB is relatively high. The reasons are related to the prominent deterioration of water and soil environment in the Yangtze River basin, the poor water quality in some tributaries, the serious eutrophication in lakes and reservoirs, and heavy metal pollution in some areas. The upper reaches of Dongting Lake and Poyang Lake also suffer from severe soil erosion and frequent geological disasters. The ecological functions of lakes and wetlands in the middle and lower reaches of the Yangtze River have deteriorated significantly. The ranks and technology gaps of the provinces and cities in YREB are obviously behind the non-YREB provinces and cities, and with a larger gap and lower rank they former have greater room for efficiency improvement.

2. The GDP and CO_2_ efficiency values of non-YREB regions exhibit a significant difference, especially for their CO_2_ efficiency value as it has a wide range from 0.2 to 1. However, the YREB provinces’ GDP and CO_2_ efficiency values are balanced, and the differences between provinces and cities within YREB are small. 

3. Non-YREB provinces and cities have a big difference in CO_2_ efficiency rank. However, YREB provinces and cities do not show a big difference in CO_2_ efficiency rank. Thus, the measures taken in the non-YREB region for low-carbon emission reduction are not effective enough.

4. Beijing, Hebei, Liaoning (except for 2015), Neimenggu, Ningxia, Qinghai, Shanghai, and Tianjin have regional rank and technical gap values of 1. Except for Shanghai (in YREB), the other provinces and cities are in non-YREB. Beijing, Shanghai, Tianjin, and other cities with very developed economies show that they have been addressing energy savings and air pollutant reduction while focusing on economic growth. The economically underdeveloped provinces of Neimenggu, Ningxia, and Qinghai have rank and technical gap values that correlate with their backward economic performance.

According to the efficiency values of different indicators, each province or city should adopt a strategy that is consistent with its actual situation. We now present some policy recommendations.

1. China’s central government should promote institutional innovation in river basin management, implement the strictest management system, and increase accountability for ecological and environmental protection. The inter-ministerial joint meeting mechanism of the Yangtze River Economic Belt that was established can also promote joint prevention and treatment of heavy chemical industry development and ecological environment. Moreover, each province should set up regional industry platforms and regional cooperation mechanisms with Shanghai, Wuhan, and Chongqing as the center. The government can integrate the industrial chain of YREB, strengthen industrial interaction among the upper, middle, and lower industry streams, and realize the coordinated development of the regional economy along the river.

2. The central government should also cultivate regional characteristic industries, promote the upgrading of provinces’ economic structure, and build green ecological industries, new technology industries, and intelligent manufacturing and service industries to solve the energy consumption and pollution problems caused by traditional industries. From the regional perspective, Jiangsu, Zhejiang, Shanghai and other provinces focus on the development of green intelligent manufacturing and advanced manufacturing, while Inner Neimenggu, Ningxia, and Qinghai can develop green energy such as solar photovoltaic, wind energy, and clean coal. From the perspective of industry, the regions’ government can target the development of high-tech industries, intelligent manufacturing, and service industries, improve the degree of industrial concentration, promote advanced industry to solve the problems of energy consumption and pollution caused by traditional industry, and ultimately achieve green and low-carbon development of the overall industrial structure. The YREB regions can take the lead in industrial upgrading and high-end manufacturing and assist in the transformation and upgrading of non-YREB regions’ industrial structure and high-quality development.

3. The central authority should also promote greater energy conservation and environmental protection technology investment and further strengthen low-carbon technology, emission reduction technology, and green energy technology. Special joint actions must be taken to clean up chemical and other pollution problems within YREB and to lay out roadmaps and timetables to solve the problems of enterprises with different pollution levels. Each province should execute ecological restoration projects along the Yangtze River and prevent atmospheric, water, and soil pollution. The YREB region can also establish multiple ecological protection mechanisms such as emission rights, carbon emission rights, energy use rights, and water rights transactions. Conversely, the non-YREB region should address technological innovation, deepen supply-side reforms, and improve the overall efficiency of regional energy conservation and emission reduction.

4. Finally, the China government can accelerate the construction of a national carbon emissions trading market system and promote a system for carbon rights exchange. In 2015, the government did propose to reduce carbon emissions by 40–45% by 2020, and thus it launched carbon rights trading in some cities. These pilot cities are Shanghai, Hubei, and Chongqing in YREB and Beijing, Tianjin, Guangdong, and Shenzhen in non-YREB. These pilot cities are going to be the model for the country’s carbon trading market system.

There are still some limitations to this study. For example, the factors behind the differences in carbon emissions between the two regions need to be further analyzed in greater depth. As a next step, future studies can consider the exploration and mining of impact factor analysis and spatial spillover effect analysis in order to add to the findings herein.

## Figures and Tables

**Table 1:** Input and output variables.

Input Variables	Output Variable	Undesirable Output Variable
Labor (lab)	GDP	CO_2_
Fixed assets (asset)		
Energy consumption (com)		

**Table 2:** Provinces’ overall efficiency score and rank.

No.	DMU	2013		2014		2015		2016	
		Rank	Score	Rank	Score	Rank	Score	Rank	Score
1	Beijing	1	1	1	1	1	1	1	1
2	Neimenggu	1	1	1	1	1	1	1	1
3	Shanghai	1	1	1	1	1	1	1	1
4	Tianjin	1	1	1	1	1	1	1	1
5	Guangdong	5	0.8991	5	0.8826	5	0.8780	6	0.8679
6	Jiangsu	6	0.8523	7	0.8170	7	0.8284	8	0.8073
7	Zhejiang	7	0.8521	8	0.8115	8	0.8043	11	0.7773
8	Hunan	8	0.8451	6	0.8344	6	0.8340	7	0.8262
9	Hainan	9	0.8223	9	0.8046	12	0.7688	14	0.7384
10	Fujian	10	0.8194	10	0.7987	9	0.7980	10	0.7776
11	Shandong	11	0.8103	11	0.7889	11	0.7691	13	0.7560
12	Hubei	12	0.7578	12	0.7459	15	0.7387	15	0.7260
13	Hebei	13	0.7475	16	0.7459	18	0.6825	18	0.6513
14	Guangxi	14	0.7460	14	0.8826	14	0.7581	12	0.7685
15	Liaoning	15	0.7411	15	0.7290	10	0.7910	1	1
16	Sichuan	16	0.7285	13	0.7402	13	0.7666	9	0.7879
17	Chongqing	17	0.7264	17	0.6997	16	0.7057	16	0.6903
18	Jilin	18	0.7236	18	0.6993	17	0.6843	17	0.6666
19	Jiangxi	19	0.6997	20	0.6580	21	0.6298	23	0.6004
20	Anhui	20	0.6827	19	0.6604	19	0.6607	19	0.6464
21	Heilongjiang	21	0.6217	21	0.6452	20	0.6303	20	0.6463
22	Henan	22	0.6216	23	0.6017	24	0.5854	24	0.5687
23	Qinghai	23	0.6068	22	0.6059	22	0.6148	21	0.6237
24	Shaanxi	24	0.6012	25	0.5814	25	0.5662	25	0.5510
25	Ningxia	25	0.5906	26	0.5602	26	0.5619	27	0.5441
26	Yunnan	26	0.5829	24	0.5910	23	0.5927	22	0.6014
27	Guizhou	27	0.5251	27	0.5300	27	0.5465	26	0.5501
28	Xinjiang	28	0.5250	28	0.5048	28	0.4734	28	0.4558
29	Shanxi	29	0.5155	29	0.4892	29	0.4675	29	0.4474
30	Gansu	30	0.5048	30	0.4784	30	0.4507	30	0.4295
Average YREB		0.7502		0.7502		0.7502		0.7284	
Average non-YREB		0.7313		0.7313		0.7313		0.7101	

Notes: The data source comes from the authors’ collection.

**Table 3:** Energy consumption, GDP, and CO_2_ efficiency scores of each province from 2013 to 2016.

DMU	2013			2014			2015			2016		
	Com	GDP	CO_2_	Com	GDP	CO_2_	Com	GDP	CO_2_	Com	GDP	CO_2_
Anhui	0.5056	0.8875	0.5056	0.4822	0.8754	0.4822	0.4371	0.8817	0.4371	0.4063	0.8758	0.4063
Beijing	1	1	1	1	1	1	1	1	1	1	1	1
Chongqing	0.8484	0.8684	0.8484	0.8241	0.8504	0.8241	0.7965	0.8567	0.7965	0.7282	0.8560	0.7282
Fujian	0.9141	0.9209	0.9141	0.8968	0.9064	0.8968	0.8982	0.9076	0.8982	0.8816	0.8941	0.8816
Gansu	0.4335	0.7745	0.4335	0.4034	0.7609	0.4034	0.3603	0.7475	0.3603	0.3267	0.7372	0.3267
Guangdong	0.9570	0.9588	0.9570	0.8509	0.9731	0.8509	0.8482	0.9740	0.8482	0.7451	0.9892	0.7452
Guangxi	0.8133	0.8785	0.8133	0.8369	0.8718	0.8369	0.8364	0.8844	0.8692	0.8280	0.8906	0.8772
Guizhou	0.2533	0.8057	0.2533	0.2632	0.8079	0.2632	0.2648	0.8187	0.2648	0.2644	0.8211	0.2644
Hainan	0.9327	0.9369	0.9327	0.9166	0.9230	0.9166	0.8876	0.8989	0.8876	0.8609	0.8779	0.8609
Hebei	0.3435	0.9558	0.3435	0.3445	0.9212	0.3445	0.3097	0.9127	0.3097	0.2988	0.8913	0.2988
Heilongjiang	0.4404	0.8482	0.4404	0.3430	0.8759	0.3430	0.3023	0.8709	0.3023	0.2441	0.8896	0.2441
Henan	0.5239	0.8382	0.5239	0.5132	0.8269	0.5132	0.4871	0.8196	0.4871	0.4652	0.8114	0.4652
Hubei	0.8204	0.8845	0.8204	0.8130	0.8784	0.8130	0.7661	0.8804	0.7661	0.7343	0.8769	0.7343
Hunan	0.8562	0.9317	0.8562	0.8566	0.9252	0.8566	0.7535	0.9448	0.7535	0.7112	0.9475	0.7112
Jiangsu	0.7692	0.9497	0.7692	0.7506	0.9306	0.7506	0.6829	0.9483	0.6829	0.6431	0.9409	0.6431
Jiangxi	0.8261	0.8519	0.8261	0.7851	0.8300	0.7851	0.7028	0.8228	0.7029	0.6347	0.8126	0.6347
Jilin	0.5012	0.9072	0.5012	0.4671	0.8958	0.4671	0.4390	0.8896	0.4390	0.4087	0.8818	0.4087
Liaoning	0.6005	0.9135	0.6005	0.5621	0.9043	0.5621	0.4275	0.9642	0.4275	1	1	1
Neimenggu	1	1	1	1	1	1	1	1	1	1	1	1
Ningxia	0.1263	0.8739	0.1263	0.1164	0.8530	0.1164	0.1049	0.8549	0.1049	0.0953	0.8427	0.0953
Qinghai	0.4422	0.8462	0.4422	0.4811	0.8425	0.4811	0.5215	0.8435	0.5215	0.5908	0.8416	0.5908
Shandong	0.5497	0.9577	0.5497	0.4948	0.9520	0.4948	0.4371	0.9589	0.3177	0.3886	0.9577	0.2623
Shanghai	1	1	1	1	1	1	1	1	1	1	1	1
Shanxi	0.1449	0.8105	0.1449	0.1252	0.7946	0.1252	0.1130	0.7811	0.1130	0.0986	0.7689	0.0986
Shaanxi	0.4063	0.8389	0.4063	0.3623	0.8310	0.3623	0.3163	0.8261	0.3163	0.2786	0.8201	0.2786
Sichuan	0.8495	0.8692	0.8495	0.8552	0.8735	0.8552	0.8775	0.8909	0.8775	0.8917	0.9023	0.8917
Tianjin	1	1	1	1	1	1	1	1	1	1	1	1
Xinjiang	0.2546	0.8054	0.2546	0.2186	0.7957	0.2186	0.1804	0.7786	0.1804	0.1529	0.7695	0.1529
Yunnan	0.5002	0.8165	0.5002	0.5294	0.8184	0.5294	0.5580	0.8163	0.5580	0.5992	0.8171	0.5992
Zhejiang	0.9200	0.9261	0.9200	0.8707	0.9094	0.8707	0.8125	0.9136	0.8125	0.7547	0.9055	0.7547

Notes: The data are from the authors’ collection.

**Table 4:** Regional rankings and technology gap values for the provinces from 2013 to 2016.

DMU	2013 Rank	2013 Total Technology Gap	2014 Rank	2014 Total Technology Gap	2015 Rank	2015 Total Technology Gap	2016 Rank	2016 Total Technology Gap
Beijing	1	1	1	1	1	1	1	1
Hebei	1	1	1	1	1	1	1	1
Liaoning	1	1	1	1	20	0.9594	1	1
Neimenggu	1	1	1	1	1	1	1	1
Ningxia	1	1	1	1	1	1	1	1
Qinghai	1	1	1	1	1	1	1	1
Shanghai	1	1	1	1	1	1	1	1
Tianjin	1	1	1	1	1	1	1	1
Guangxi	9	0.9973	9	0.9969	8	0.9992	1	1
Jilin	10	0.9969	10	0.9941	10	0.9965	12	0.9958
Shaanxi	11	0.9967	13	0.9918	13	0.9866	14	0.9828
Shandong	12	0.9927	11	0.9929	9	0.9965	11	0.9976
Zhejiang	13	0.9920	17	0.9720	16	0.9708	19	0.9562
Fujian	14	0.9854	12	0.9919	12	0.9947	13	0.9947
Hainan	15	0.9835	14	0.9906	11	0.9955	10	0.9986
Gansu	16	0.9770	16	0.9778	15	0.9753	17	0.9741
Xinjiang	17	0.9744	15	0.9785	14	0.9782	16	0.9791
Heilongjiang	18	0.9727	20	0.9437	21	0.9343	21	0.9178
Henan	19	0.9716	18	0.9696	18	0.9686	18	0.9680
Sichuan	20	0.9696	21	0.9397	19	0.9626	20	0.9432
Shanxi	21	0.9676	19	0.9683	17	0.9701	15	0.9814
Chongqing	22	0.9603	22	0.9205	22	0.9216	22	0.9011
Jiangxi	23	0.9330	25	0.8947	24	0.9007	25	0.8822
Yunnan	24	0.9317	23	0.9010	23	0.9064	23	0.8900
Hubei	25	0.9297	26	0.8840	25	0.8903	27	0.8692
Guizhou	26	0.8993	28	0.8799	27	0.8831	26	0.8768
Guangdong	27	0.8991	27	0.8826	28	0.8780	28	0.8679
Anhui	28	0.8724	29	0.8498	29	0.8608	29	0.8578
Jiangsu	29	0.8523	24	0.8954	26	0.8875	24	0.8890
Hunan	30	0.8451	30	0.8344	30	0.8340	30	0.8262
Average YREB	0.9259		0.9065		0.9107		0.8992	
Average non-YREB	0.9850		0.9831		0.9807		0.9820	

Notes: The data are from the authors’ collection.

**Table 5:** Wilcoxon test score of the average technology efficiency gap.

Year	Ave. Gap of YREB	Ave. Gap of Non-YREB	Wilcoxon Test Score
2013	0.925952	0.984987	0.0034 ***
2014	0.906488	0.983087	0.0019 ***
2015	0.910705	0.980675	0.0033 ***
2016	0.899237	0.981993	0.0018 ***

Notes: *** significant confidence interval of 0.01 (two-tailed test). The data in Table 5 are calculated with SAS 9.4 software (SAS Institute Inc., Cary, NC, USA).
